# Recent advances on the regulation of bacterial biofilm formation by herbal medicines

**DOI:** 10.3389/fmicb.2022.1039297

**Published:** 2022-11-08

**Authors:** Meimei Zhang, Wenyu Han, Jingmin Gu, Cao Qiu, Qiujie Jiang, Jianbao Dong, Liancheng Lei, Fengyang Li

**Affiliations:** ^1^State Key Laboratory for Zoonotic Diseases, Key Laboratory of Zoonosis Research, Ministry of Education, College of Veterinary Medicine, Jilin University, Changchun, China; ^2^Jiangsu Co-Innovation Center for the Prevention and Control of Important Animal Infectious Diseases and Zoonoses, Yangzhou University, Yangzhou, China; ^3^Jilin Animal Disease Control Center, Changchun, China; ^4^Department of Veterinary Medical, Shandong Vocational Animal Science and Veterinary College, Weifang, China

**Keywords:** biofilm formation, traditional Chinese medicine, anti-biofilm agents, quorum sensing, second messenger

## Abstract

Biofilm formation is a fundamental part of life cycles of bacteria which affects various aspects of bacterial-host interactions including the development of drug resistance and chronic infections. In clinical settings, biofilm-related infections are becoming increasingly difficult to treat due to tolerance to antibiotics. Bacterial biofilm formation is regulated by different external and internal factors, among which quorum sensing (QS) signals and nucleotide-based second messengers play important roles. In recent years, different kinds of anti-biofilm agents have been discovered, among which are the Chinese herbal medicines (CHMs). CHMs or traditional Chinese medicines have long been utilized to combat various diseases around the world and many of them have the ability to inhibit, impair or decrease bacterial biofilm formation either through regulation of bacterial QS system or nucleotide-based second messengers. In this review, we describe the research progresses of different chemical classes of CHMs on the regulation of bacterial biofilm formation. Though the molecular mechanisms on the regulation of bacterial biofilm formation by CHMs have not been fully understood and there are still a lot of work that need to be performed, these studies contribute to the development of effective biofilm inhibitors and will provide a novel treatment strategy to control biofilm-related infections.

## Introduction

Biofilm is a self-protective state formed by bacteria to adapt to the poor living environment. It is a microbial community attached to biotic or abiotic surfaces and wrapped by self-produced extracellular polymeric matrix (EPS) that contains extracellular polysaccharides, nucleic acids (extracellular DNA and extracellular RNA), amyloid proteins, lipids, and many other biomolecules (Karygianni et al., 2020). All bacterial species can form biofilm under suitable conditions, and actually it is estimated that more than 90% of microorganisms exist in the form of biofilm (Costerton et al., 1999). Bacteria in biofilms are physiologically distinct from their planktonic cell state which makes them tolerant to harsh conditions and tolerance to antibacterial treatments such as antibiotics (Roy et al., 2018; Hawas et al., 2022). In clinical settings, biofilm formation of pathogens causes persist infections and biofilm-related infections are becoming increasingly difficult to treat due to tolerance to antibiotics which poses a great threat to human health. It is estimated that approximately 65%–80% of bacterial infections in humans are associated with biofilm formation (Chen et al., 2010; Bjarnsholt et al., 2018). Thus, it is urgent to develop effective and robust strategies to control biofilm formation of pathogens.

**Figure 1:**
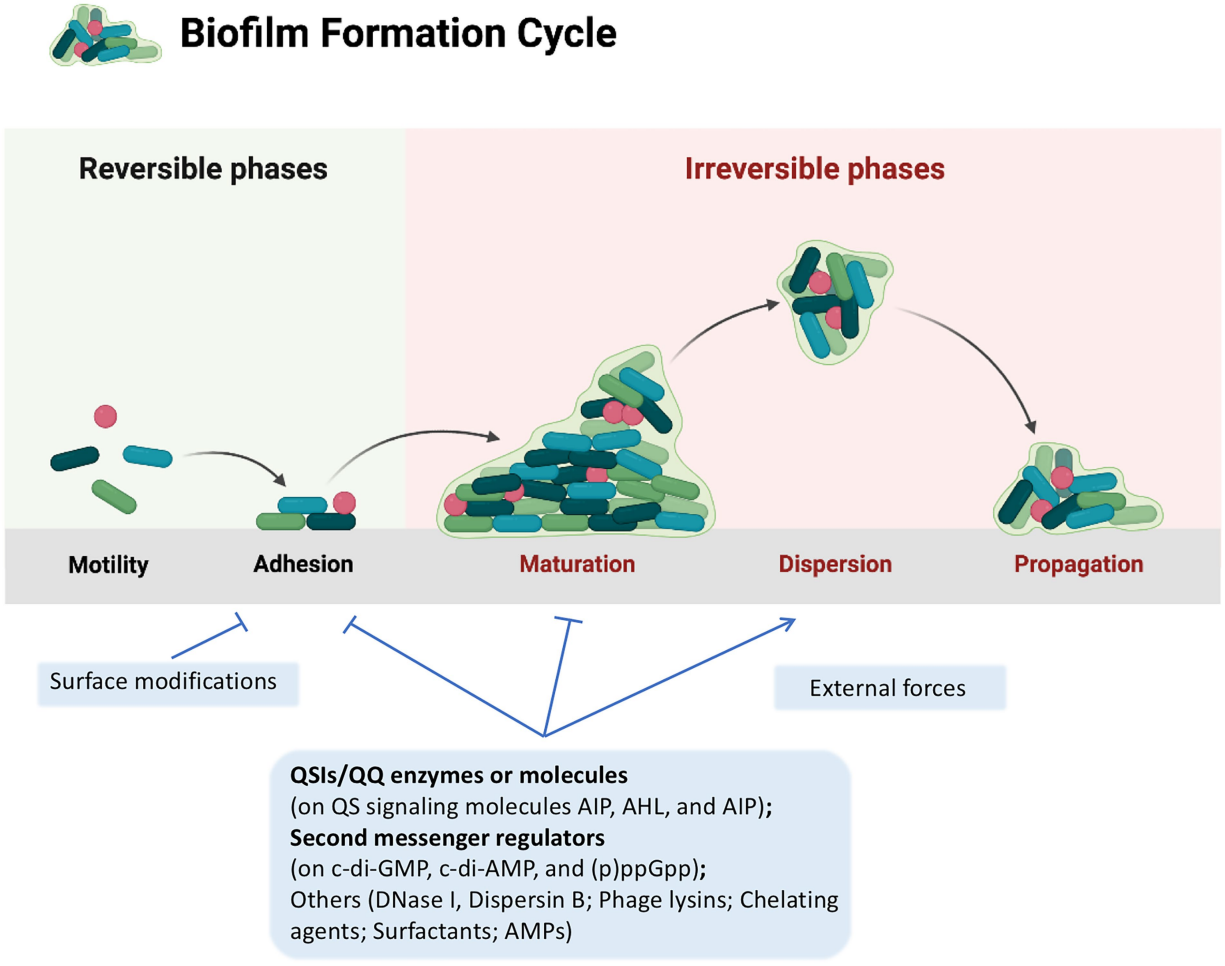
A typical biofilm cycle and the strategies to control biofilm formation. A typical biofilm formation consists of five stages: (i) reversible attachment to surface; (ii) irreversible attachment to surface; (iii) microcolony formation; (iv) maturation of biofilm; and (v) biofilm dispersal. Strategies for combating bacterial biofilms are classified into three main categories: (i) changing the properties of susceptible surfaces to prevent biofilm formation; (ii) regulating signaling pathways to inhibit biofilm formation; and (iii) applying external forces to eradicate the biofilm, which are displayed in light blue rectangles. Strategies discussed in this review are presented in bold. Lines with arrow head, positive regulation; Lines with stops, negative regulation. This figure was created with BioRender.com.

Strategies for combating bacterial biofilms have been classified into three main categories: (i) changing the properties of susceptible surfaces to prevent biofilm formation; (ii) regulating signaling pathways to inhibit biofilm formation; (iii) applying external forces to eradicate the biofilm (Yin et al., 2021; Figure 1). Besides the development of novel biofilm-resistant materials and application of physical forces to eradicate biofilms, most of the researchers focus on investigating the regulatory signaling pathways of biofilm formation including bacterial quorum sensing (QS) system and nucleotide-based second messengers cyclic dimeric guanosine monophosphate (c-di-GMP), cyclic dimeric adenosine monophosphate (c-di-AMP), cyclic guanosine monophosphate (cGMP), cyclic adenosine monophosphate (cAMP) and guanosine tetraphosphate ((p)ppGpp; Wu et al., 2015; Yin et al., 2021), and several kinds of anti-biofilm agents have been discovered so far, including Quorum Sensing Inhibitors (QSIs) such as quercetin which dampens QS signaling (Ouyang et al., 2016), and nitric oxide (NO)-generating agents such as sodium nitroprusside (SNP) that restricts c-di-GMP signaling (Barraud et al., 2009). Other anti-biofilm agents targeting bacterial adhesion and disruption of extracellular DNA have also been identified recently, such as Dispersin B which cleaves the major EPS polysaccharide poly-β 1,6-N-acetylglucosamine, and Deoxyribonuclease I which degrades extracellular DNA present in the EPS (Kaplan et al., 2003; Qin et al., 2007).

Traditional Chinese medicine (TCM) is one of the oldest healing systems which includes herbal medicine, acupuncture, moxibustion, massage, food therapy, and physical exercise, and have been used for a long history in China against various diseases (Tang et al., 2008). Many TCMs are derived from natural herbs and Chinese herbal medicines (CHMs) are important component of TCMs (Liu et al., 2021). CHMs are usually a mixture of herbal plants or extracts which comprise hundreds of different constituents with widely differing physiochemical properties (Tang et al., 2008). As such, roots, stems, leaves and/or fruits of diverse herbs species are commonly used in CHMs. The standardized formulae of CHMs are now commonly used as tablets, capsules, and even ampoules as well as the traditional decoctions of individualized prescriptions (Tang et al., 2008; Kong et al., 2009). As natural active drugs, CHMs have the advantages of abundant resources, higher safety, and lower toxicity compared with chemically synthesized drugs (Flower et al., 2015; Liao et al., 2022). However, due to the complex composition of CHMs, the large-scale application of TCMs is limited. Thus, more and more researchers have shifted their research focus to the identification and clarification of the antibacterial mechanisms of active components from CHMs, many of which exert anti-infection effect through inhibition of bacterial biofilm formation (Liu et al., 2011; Packiavathy et al., 2014). In exploring their antibacterial mechanisms, it was found that different chemical classes of CHMs metabolites, including flavonoids, terpenoids, phenols, organic acids, alkaloids and their derivatives, can inhibit bacterial biofilm formation by regulating bacterial QS system and nucleotide-based second messengers. In this review, we describe the research progresses of CHMs that act on bacterial QS system and second messengers in terms of bacterial biofilm formation, and to provide evidence of the potential of CHMs for the treatment and/or control of biofilms-associated infections and, in this way, encourage more and more advanced research on this area.

## Quorum sensing

Quorum sensing (QS) is a bacterial communication system that plays a pivotal role in regulating bacterial biofilm formation (Irie and Parsek, 2008). QS is driven by signaling molecules in a density-dependent manner that contributes to a variety of biological functions, such as virulence factor secretion (Singh and Ray, 2014; Hernández-Ramírez et al., 2020), swimming/swarming motility (Daniels et al., 2004; Yang and Defoirdt, 2015), and bioluminescence (Nealson et al., 1970; Zhao et al., 2016). Various signaling molecules have been identified in bacteria so far, including N-acyl-homoserine lactone (AHL), autoinducing peptide (AIP), autoinducer-2 (AI-2), AI-3/epinephrine/norepinephrine signaling molecules, the diffusible signal factor (DSF), and 2-(2-hydroxyphenyl)-thiazole-4-carbaldehyde (Irie and Parsek, 2008; Dickschat, 2010; Lee et al., 2013; Zhou et al., 2017). Among these molecules, AHL, AIP, and AI-2 are most widely studied. These different signaling molecules mediate different types of QS systems (Reading and Sperandio, 2006). While the QS system of most Gram-negative bacteria is the LuxI/LuxR type self-induction system that uses AHL as signaling molecule (Parsek and Greenberg, 2000), the QS system of Gram-positive bacteria is mediated by the small molecule peptide AIP (Kleerebezem et al., 1997). Moreover, there is a QS system that exists in both Gram-negative and Gram-positive bacteria, the LuxS/AI-2 type signaling system which uses AI-2 as the system’s signaling molecule for information exchange between bacterial species (Chen et al., 2002; Camilli and Bassler, 2006).

The regulatory mechanism of the bacterial QS system has been extensively studied. It has been found that the system can be targeted for the development of antibacterial inhibitors, and such inhibitors are called Quorum Sensing Inhibitors (QSIs; Chaieb et al., 2022). In addition to common antimicrobial peptides and antibiotics, many natural active substances extracted from TCMs and plants are also QSIs that can play an important role in the regulation of bacterial biofilm formation. The mechanisms of QSIs in blocking QS pathway are broadly classified into three types: (i) inhibition of signaling molecules synthesis; (ii) promotion of signaling molecules degradation; and (iii) competition with signaling molecules for receptor proteins binding (Zhou et al., 2020). Table 1 shows TCMs metabolites and their derivatives which displayed anti-bacterial biofilm formation *via* QS in the literatures, as well as their targets.

**Table 1:** Different classes of anti-biofilm TCMs metabolites and their mechanisms of action *via* bacterial QS system.

TCMs metabolites	Main plant origin	Mechanism of action	Target bacteria	Reference
**Flavonoids**				
Flavanones	Orange	Inhibits the production of AHL	*Yersinia enterocolitica*	Truchado et al. (2012)
Quercetin	*Usnea longissimi*	Reducing the expression levels of *lasI*, *lasR*, *rhlI* and *rhlR*; Competes with AHL for receptor protein	*Chromobacterium violaceum*; *Pseudomonas aeruginosa*	Gopu et al. (2015), Ouyang et al. (2016)
Curcumin	*Curcuma longa*	Competes with AHL for receptor protein LasR and LuxR	*P. aeruginosa*	Shukla et al. (2020)
Baicalin	*Scutellaria baicalensis*	Suppression of QS regulatory genes *agrA*, RNAIII and *sarA*; Inhibits the production of AI-2; binds to LuxS	*Staphylococcus aureus*; *Streptococcus saprophyticus*; APEC	Chen Y. et al. (2016), Peng et al. (2019), Wang et al. (2019), Meng et al. (2022)
Kaempferol	*Kaempferia galanga L*	Binds to LuxS;inhibits the production of AI-2	*Lactobacillus reuteri S. aureus*	Ming et al. (2017, 2022)
Fisetin	*Cotinus coggygria*	Inhibits the production of AI-2	*S. aureus*; *Streptococcus dysgalactiae*	Dürig et al. (2010)
**Terpenoids**				
Sesquiterpene lactone	*Magnoliaceae*	Inhibits the production of AHL	*P. aeruginosa*	Amaya et al. (2012)
Carvacrol	Clove	Integration with ExpI/ExpR	*Pectobacterium*	Joshi et al. (2016)
Eugenol	Passion fruit	Integration with ExpI/ExpR	*Pectobacterium*	Joshi et al. (2016)
Sclareol	*Salvia miltiorrhiza Bge.*	Blocking AgrA from binding to DNA or activating *agrA* after phosphorylation	*S. aureus*	Iobbi et al. (2021)
Manool	*Salvia miltiorrhiza Bge.*	Blocking AgrA from binding to DNA or activating *agrA* after phosphorylation	*S. aureus*	Iobbi et al. (2021)
Andrographolide	*Andrographis paniculata*	Inhibits the activity of AI-2; decreases the expression level of *arg* gene and the activity of *arg* promoter P2	*Escherichia coli*	Guo et al. (2014), Yu et al. (2022)
**Phenols**				
Catechin	*Combretum albiflorum*	Reduction of the expression of QS controlled virulence factors	*P. aeruginosa*	Vandeputte et al. (2010)
Hamamelitannin	*Hamamelis virginiana*	Suppression of QS regulatory RNAIII	*S. aureus*	Kiran et al. (2008)
Syringic acid	Oak	Suppression of QS regulatory genes *agrD* and *agrA*	*Staphylococcus epidermidis*	Minich et al. (2022)
Resveratrol	*Veratrum album*	Suppression of QS regulatory genes *agrA*, *agrB*, *agrC*, *hld* and *sarA*	*S. aureus*	Qin et al. (2014)
Ursolic acid	*Prunella vulgaris L.*; *Ilex rotunda Thunb*	Suppression of QS regulatory genes *agrA*, *agrB*, *agrC*, *hld* and *sarA*	*S. aureus*	Qin et al. (2014)
Tea polyphenols (Epigallocatechin gallate)	Green tea (*Camellia sinesis*)	Regulation of AI-2 synthesis; reduction of C4-AHL production	*S. aureus*; *Stenotrophomonas maltophilia*; *Streptococcus mutans*; *P. aeruginosa*	Dürig et al. (2010), Vidigal et al. (2014), Zhang et al. (2014), Wu et al. (2018), Hao et al. (2021)
Zingerone	Ginger	Interference with the ligand receptor interaction with QS receptors (TraR, LasR, RhlR and PqsR)	*P. aeruginosa*	Kumar et al. (2015)
**Organic acids**				
Gallic acid	Green tea (*Camellia sinesis*); *Libidibia ferrea*	Downregulates of the expression of *gtfB*, *gtfC* and *gtfD* genes; inhibits expression of *pgaABCD*	*Streptococcus pyogenes; E. coli*; *P. aeruginosa*	Kang et al. (2018), Albutti et al. (2021), Passos et al. (2021)
Vanillic acid	Vanilla beans	Inhibits the production of AHL	*C. violaceum*; *Aeromonas hydrophila*	Deryabin et al. (2019)
**Alkaloids**				
Berberine	*Coptis chinensis*	Suppression of QS regulatory gene *agrA*	*S. aureus*	Gao et al. (2021)
Matrine	*Sophora alopecuroides*, broad bean roots and *Sophora flavescens*	Inhibits the activity of AI-2	*E. coli*; *S. epidermidis*; *P. aeruginosa*	Jia et al. (2019), Pourahmad Jaktaji and Koochaki (2022)
**Others**				
Halogenated Furanones	*Delisea pulchra*	Competes with AHL for receptor protein; accelerates folding of LuxR	*Vibrio harzianus’*	Rabin et al. (2013), Reuter et al. (2016)
Trans-anethole	Anise	Binds to LasR regulatory proteins	*P. aeruginosa*	Hançer Aydemir et al. (2018)
Diallyl disulfide	Garlic	Inhibites virulence factors including exonuclease LasA, elastase LasB, lectins LecA and LecB	*P. aeruginosa*	Li et al. (2018)
Esculetin	Sieve bean	Disturbs QS	*S. aureus*; *E. coli*; *Salmonella typhimurium*; *P. aeruginosa*	Girennavar et al. (2008), Dürig et al. (2010)
Furocoumarins	Sieve bean	Disturbs QS	*S. aureus*; *E. coli*; *S. typhimurium*; *P. aeruginosa*	Girennavar et al. (2008), Dürig et al. (2010)
*Piper betle* extract	*Piper betle*	Inhibits the production of AHL	*P. aeruginosa*	Siddiqui et al. (2012)

TCMs, Traditional Chinese medicines; AHL, N-acyl-homoserine lactone; QS, Quorum sensing; AI-2, Autoinducer-2; APEC, Avian pathogenic *E. coli*.

### TCMs that inhibit quorum sensing

#### Flavonoids

**Figure 2:**
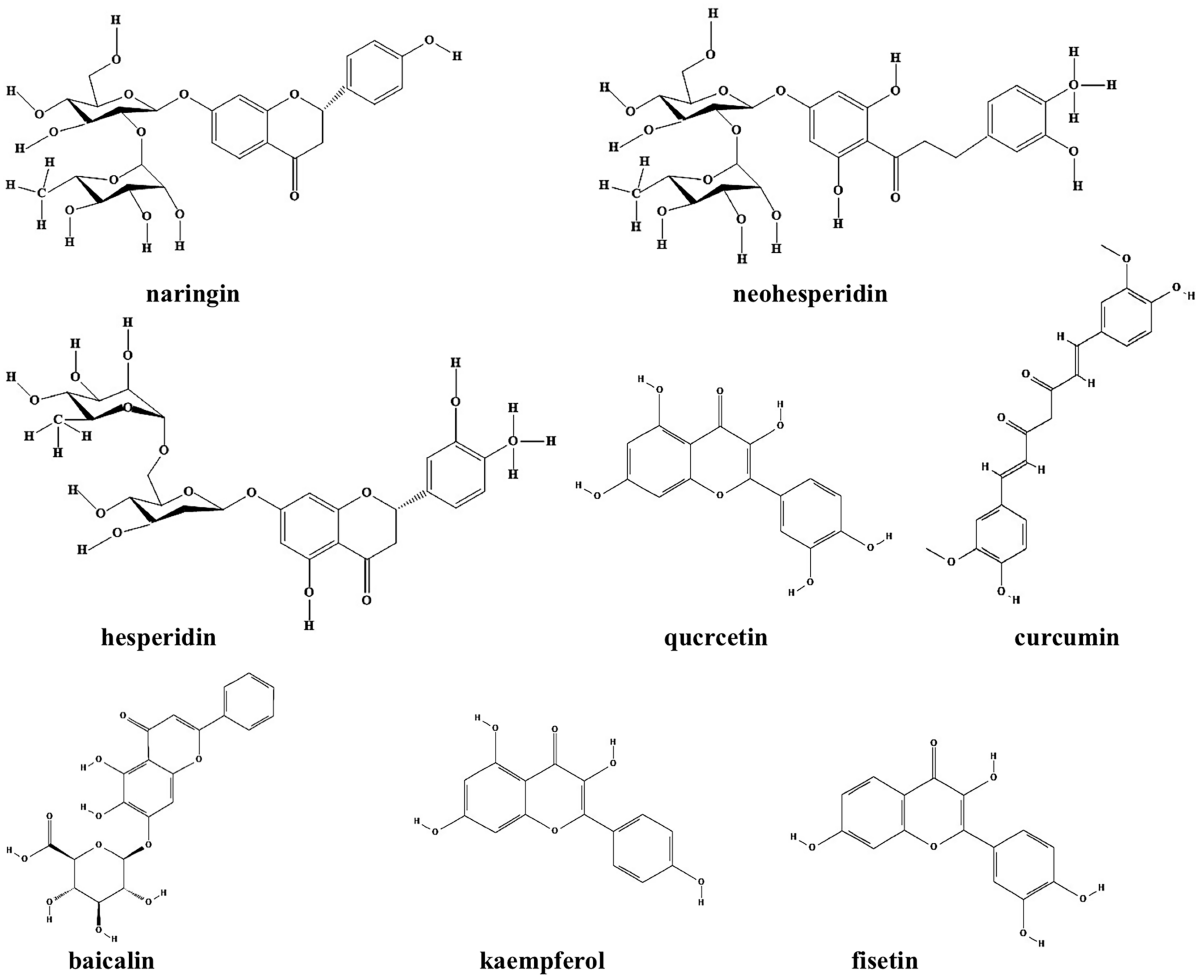
Chemical structures of the different flavonoids that inhibit biofilm formation *via* QS. ChemDraw software has been utilized to draw the chemical structures of the molecules.

Flavonoids refer to a series of chemical compounds with two variable phenolic structure and many of them show various bioactive functions including antioxidant, antiviral, antibacterial, and anti-inflammation (Chu et al., 2015; Lee et al., 2018; Table 1; Figure 2). Plants are rich in flavonoids and many of which have been utilized as TCMs for a long period, such as quercetin leaves (Ouyang et al., 2016), Pericarpium *Citri Reticulatae* (Ma et al., 2021), and *Scutellaria baicalensis* (Chen Y. et al., 2016). Clinical studies have shown that flavonoids can protects gut microbiota from dysbiosis (Klinder et al., 2016), but whether this is through QS signaling is still unknown. Common flavonoids discovered so far including flavanone, quercetin, curcumin, baicalin, kaempferol, and fisetin, all of which exhibit different degrees of anti-biofilm activity *via* bacterial QS signaling (Table 1; Figure 1).

**Figure 3:**
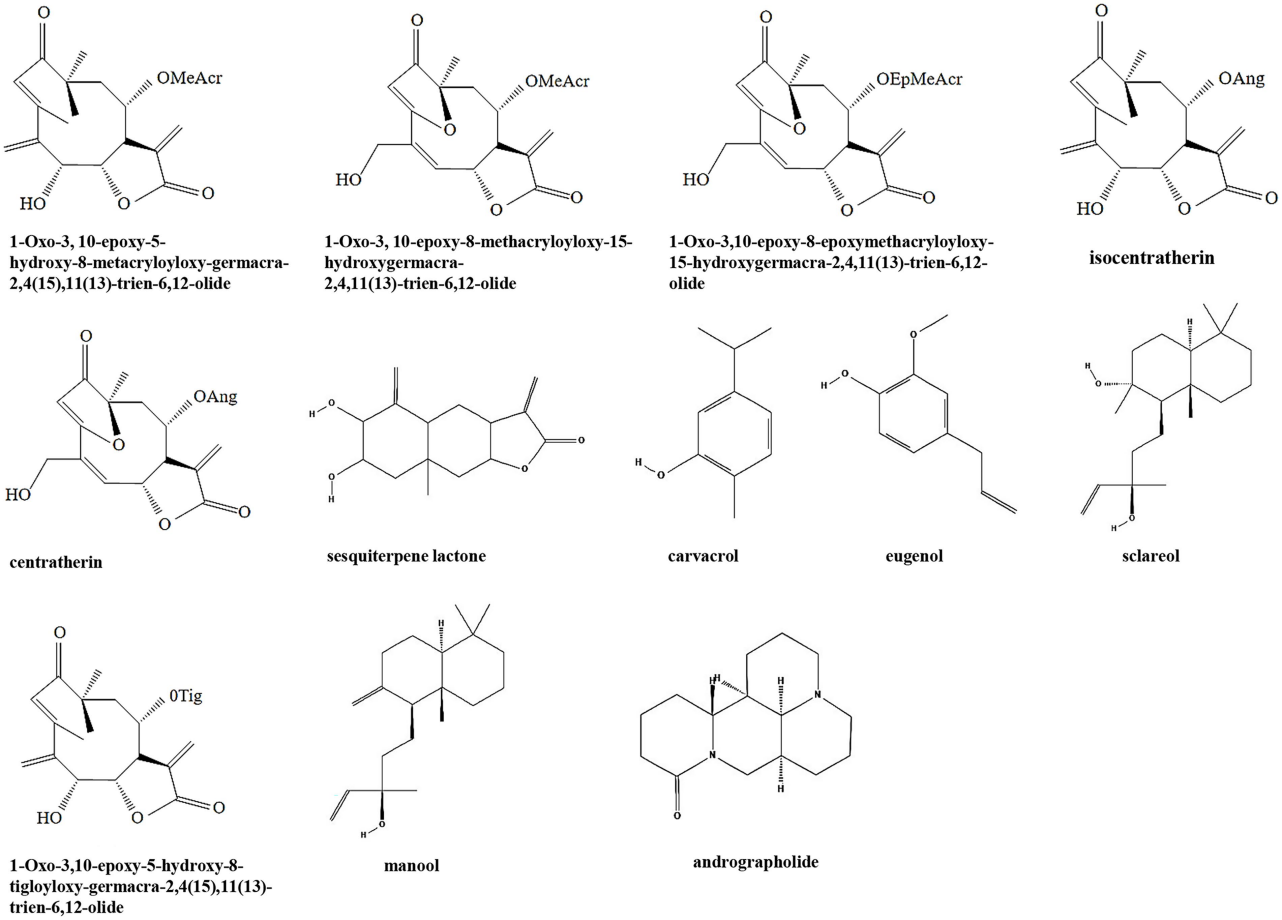
Chemical structures of the different terpenoids that inhibit biofilm formation *via* QS. ChemDraw software has been utilized to draw the chemical structures of the molecules.

The glycosylated flavanones naringin, neohesperidin, and hesperidin extracted from orange reduce the activity of *Yersinia enterocolitica* and inhibit its biofilm formation by interfering with the production of the signaling molecule AHL of QS system (Truchado et al., 2012). These flavanones downregulate the expression of genes involved in the synthesis of AHL (*yenI* and *yenR*) to impair QS signaling and biofilm formation. *In vivo*, naringin and hesperidin protects mice from endotoxin shock through inhibition of bacterial numbers and inflammatory cytokine release (Kawaguchi et al., 2004a,b).

Similarly, quercetin, another flavonoid from *Usnea longissimi*, inhibit the biofilm formation of diverse bacteria species including *Pseudomonas aeruginosa* (Ouyang et al., 2016), *Chromobacterium violaceum* (Skogman et al., 2016), and *Klebsiella pneumoniae* (Gopu et al., 2015) through QS signaling. Quercetin, although not affecting the growth of *P. aeruginosa*, significantly inhibit the production of biofilm and virulence factors by downregulation of the expression levels of *lasI*, *lasR*, *rhlI*, and *rhlR* (Ouyang et al., 2016). It further demonstrates that quercetin inhibit QS *via* binding with LuxI-type AHL synthases and/or LuxR-type AHL receptor proteins (Deryabin et al., 2019). *In vivo*, quercetin supplementation reduces the number of pathogenic species including *Enterococcus*, *Neisseria* and *Pseudomonas* and increases the number of non-pathogenic *Streptococcus* sp. and oral microbiome diversity (Mooney et al., 2021).

Moreover, curcumin from *Curcuma longa* also reduce the ability of *P. aeruginosa* to form biofilms and inhibit virulence factors expression. Curcumin binds to both LasR and LuxR that leads to the inactivation of these proteins and reduction in biofilm formation (Shukla et al., 2020). In a clinical study, curcumin treatment significantly diminishes the severity of dyspepsia and eradication of *Helicobacter pylori* in patients, indicating that curcumin can be used as a candidate drug for the treatment of functional dyspepsia (Panahi et al., 2021).

Baicalin, another flavonoid isolated from the root of *Scutellaria baicalensis*, downregulates the gene expression of *Staphylococcus aureus* QS regulators *agrA*, RNA III and *sarA* and *ica* to inhibit biofilm formation, leading to increased vancomycin permeability (Chen Y. et al., 2016). Wang et al. (2019) further demonstrated that the reduction of biofilm formation by baicalin was achieved by inhibiting the MsrA efflux pump and the Agr system in *Streptococcus saprophyticus*. Moreover, baicalin also inhibits QS signaling molecule AI-2 and the expression of virulence genes in avian pathogenic *Escherichia coli* (APEC; Peng et al., 2019). *In vivo*, baicalin significantly reduces APEC colonization and increases the abundance of short chain fatty acid (SCFA)-producing bacteria of gut microbiota to alleviate lung injury (Peng et al., 2021b).

Furthermore, *in silico* analyzation by molecular docking reveales the binding mode of four natural products, norathyriol, mangiferin, baicalein, kaempferol and baicalin, to LuxS. All of these products show good binding ability to LuxS and inhibit the production of AI-2 (Meng et al., 2022). In addition, kaempferol extracted from *Kaempferia galanga L.* could also reduce the biofilm formation of *S. aureus* by inhibit the activity sortase A and the expression of adhesion-related genes (Ming et al., 2017). This is also the case for fisetin, a compound extracted from *Cotinus coggygria*, which dramatically inhibit biofilm formation of both *S. aureus* and *Streptococcus dysgalactiae via* a similar mechanism (Dürig et al., 2010).

### Terpenoids

Terpenoids are a class of secondary metabolites that have the general formula of (C_5_H_8_) n. According to the number of isoprene or isopentane (C_5_H_8_), terpenoids and their derivatives are divided into several subclasses including monoterpenes, sesquiterpenes, diterpenes, triterpenes, tetriterpenes, and polyterpenes (Zhuang and Chappell, 2015). Terpenoids are widely distributed in nature and many of them play a wide range of pharmacological effects as TCMs, such as antiparasitic and antibacterial effects. Many terpenoids including sesquiterpene lactones, carvacrol, eugenol, sclareol, manool, and andrographolide have been discovered with anti-biofilm activity (Table 1; Figure 2). It is been shown that six sesquiterpene lactones, three of the goyazensolide-type and three of the isogoyazensolide-type extracted from *Centratherum punctatum*, inhibited biofilm formation of *P. aeruginosa* by downregulation of QS signaling molecule AHL and inhibit bacterial growth in a concentration dependent manner (Amaya et al., 2012), but the detailed molecular mechanisms still need to be investigated.

Carvacrol and eugenol, which are commonly isolated from clove and passion fruit, respectively, and utilized in essential oils, could also specifically interfere with the QS system of *Pectobacterium*. By constructing homology models for high serine lactone synthase (ExpI) or regulatory proteins (ExpR) and performing molecular docking simulation tests, carvacrol and eugenol have the ability to bind ExpI/ExpR, which in turn leads to decreased accumulation of the intracellular QS signaling molecule AHL and inhibit biofilm formation (Joshi et al., 2016; Deryabin et al., 2019). Moreover, eugenol inhibit the formation of *Acinetobacter baumannii* biofilms and disrupt biofilm structure by downregulation of the transcription of genes involved in biofilm formation (Karumathil et al., 2016). *In vivo* studies demonstrate that carvacrol inhibits the colonization of several pathogens, including *Campylobacter jejuni* (Mousavi et al., 2020), *S. typhimurium* (Kortman et al., 2014), and *Listeria monocytogenes* (Silva et al., 2015), to host cells and thus protest host from infections. Similarly, eugenol can also inhibit the colonization of *S. typhimurium* and restricts host inflammation *(*Zhao et al., 2022).

The labdane diterpenoids sclareol and manool from *Salvia tingitana* are considered potential QSIs against methicillin-resistant *S. aureus* (MRSA). They can inhibit MRSA biofilm formation and virulence factor expression by prevention of the activation of AgrA upon binding or phosphorylation of the helper gene regulator AgrA to DNA (Iobbi et al., 2021). Guo et al. investigated the effect of andrographolide, the main active ingredient of *Andrographis paniculata*, on the pathogenies of APEC O78. They found that andrographolide significantly decrease the lactate dehydrogenase release, F-actin cytoskeleton polymerization, and bacterial adhesion to chicken type II pneumocytes by inhibiting the expression of QS signaling molecule AI-2 and virulence factors (Guo et al., 2014). However, study also showed that andrographolide had no effect on the production of AI-2, but significantly decreased the expression level of *arg* gene and the activity of *arg* promoter P_2_, leading to inhibition of the biofilm formation and virulence of *L. monocytogenes* (Yu et al., 2022).

### Phenols

**Figure 4:**
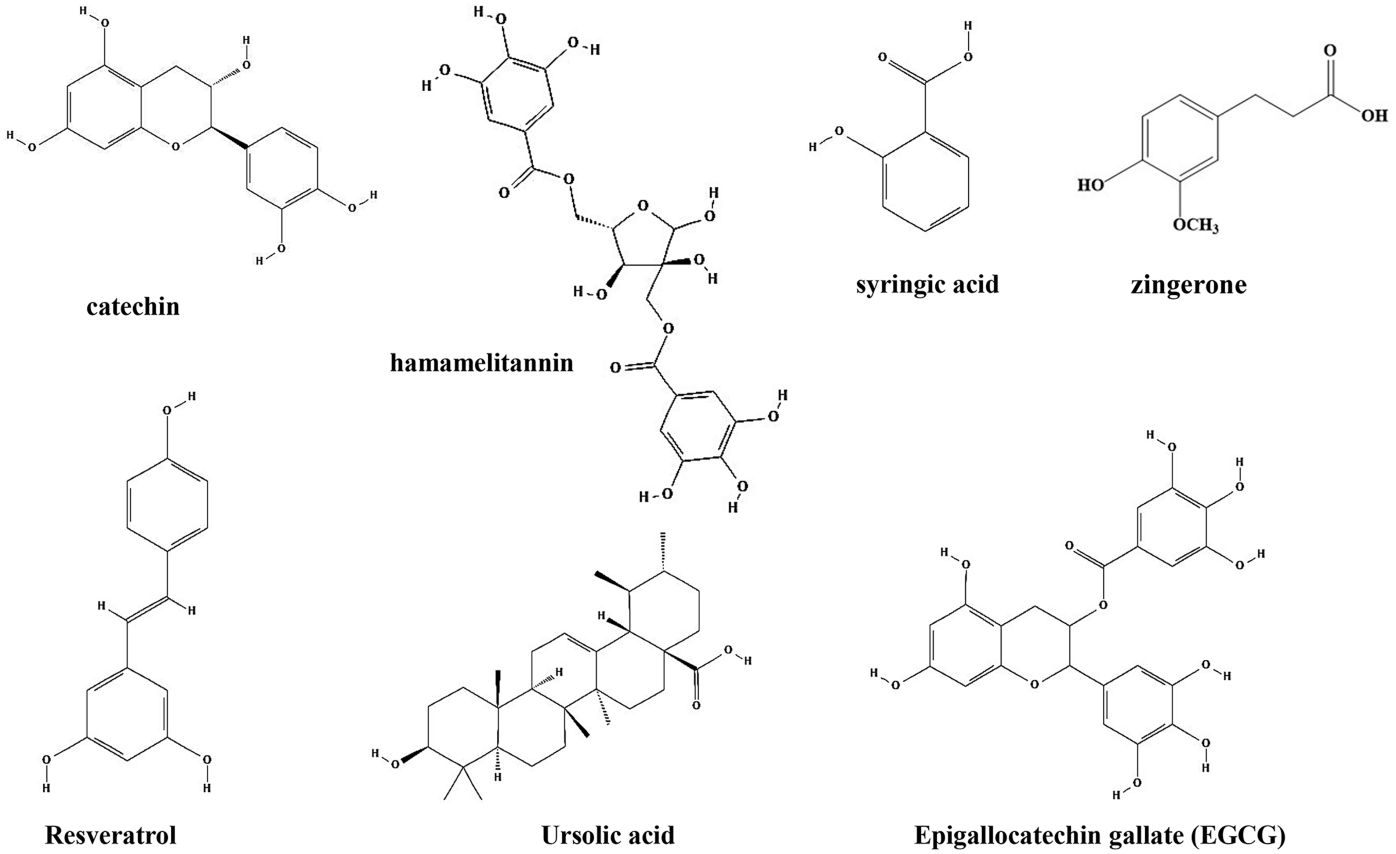
Chemical structures of the different phenols that inhibit biofilm formation *via* QS. ChemDraw software has been utilized to draw the chemical structures of the molecules.

Plant phenols are found in the leaves, shells, pulp and seed coat of plants, and are second only to cellulose, hemicellulose and lignin in content. Plant phenols have a long history of medical applications and have been shown to have strong antioxidant activity, effective in preventing chronic diseases such as hyperglycemia (Westfall et al., 2018), hyperlipidemia (Yazdanparast et al., 2008), cardiovascular and cerebrovascular diseases (Wu et al., 2010), as well as reducing cancer risk (Cesmeli et al., 2021). Common plant phenols such as catechin, hamamelitannin, syringic acid, ursolic acid, zingerone, resveratrol, and tea polyphenols have been shown to inhibit the formation of biofilm by bacteria (Table 1; Figure 4).

Catechin, one of the phenols isolated from *Combretum albiflorum* leaves and bark extracts, inhibit the biofilm formation and pathogenesis by reduction of the expression of QS controlled virulence factors in *P. aeruginosa* (Vandeputte et al., 2010). The use of RhlR-and LasR-based biosensors indicated that catechin might interfere with the perception of the QS signal N-butanoyl-L-homoserine lactone by RhlR, thereby leading to a reduction of the production of QS factors. *In vivo* studies showed that catechin can promote the proliferation of beneficial intestinal bacteria and regulate the balance of intestinal flora to relieve the inflammatory bowel disease (Fan et al., 2017). Hamamelitannin, a polyphenolic natural product found in the bark of *Hamamelis virginiana*, has no effect on staphylococcal growth *in vitro*, but reduce biofilm formation by inhibiting the QS regulator RNA III (Kiran et al., 2008). Moreover, several synthetic hamamelitannin analogs have been identified as antibiotic potentiators for *S. aureus* treatment (Vermote et al., 2016). Hamamelitannin increases the susceptibility of *S. aureus* to antibiotic treatment *in vivo Caenorhabditis elegans* and mouse mammary gland infection models (Brackman et al., 2016). Syringic acid, which is also a phenolic compound isolated from oak bark lignin, reduce biofilm formation up to 80% and EPS up to 55% by downregulation of mRNA expression of two genes of the QS system, *agrD* and *agrA* in *Staphylococcus epidermidis* (Minich et al., 2022). Moreover, inhibition of biofilm formation by interfering with the QS system is also observed by treatment with resveratrol (extracted from *Veratrum album*, a plant of *Liliaceae*) and ursolic acid (found in the whole grass of *Prunella vulgaris L.*, a labiatae plant, and the leaves of *Ilex rotunda Thunb*), upon which the expressions of genes related to the QS system (*agrA*, *agrB*, *agrC*, *hld* and *sarA*) are downregulated (Qin et al., 2014). Similar to catechin, resveratrol and ursolic acid have also shown protective effects on gut microbiota *in vivo* (Cai et al., 2020; Peng et al., 2021a).

Investigation of the molecular mechanism also identified several phenolic compounds that interacts with QS signaling molecules. Zingerone, which is mainly found in root of ginger (*Zingiber officinale*), reduces the ability of *P. aeruginosa* to form biofilms and inhibits virulence factors expression by competing with signaling molecules for receptor proteins (TraR, LasR, RhlR and PqsR), thereby blocked the QS signaling (Kumar et al., 2015). Of note, zingerone effectively reduced *P. aeruginosa* biofilm-associated murine acute pyelonephritis (Sharma et al., 2020), suggesting it is a potential effective therapeutic agent for clinical application. Zhang et al. investigated the effects of citral, cinnamaldehyde, and tea polyphenols on the formation of mixed biofilms of foodborne *S. aureus* and *Salmonella enteritidis*. The results showed that citral, cinnamaldehyde and tea polyphenols could significantly inhibit the formation of mixed biofilms. Interestingly, while citral could reduce the synthesis of AI-2, cinnamaldehyde and low concentrations of tea polyphenols increased AI-2 synthesis (Zhang et al., 2014). Similarly, Epigallocatechin gallate (EGCG, tea polyphenol), which is present in green tea, also showed anti-biofilm and anti-infection activities by *Stenotrophomonas maltophilia* and *P. aeruginosa* by reduction of C4-AHL production (Vidigal et al., 2014; Hao et al., 2021). In mice, these compounds protect mice from infections by different pathogens, including methicillin-resistant *S. aureus* (Long et al., 2019), *H. pylori* (Muhammad et al., 2015; Deng et al., 2022), and *S. typhimurium* (Wang et al., 2021; Zhao et al., 2021).

### Organic acids

**Figure 5:**
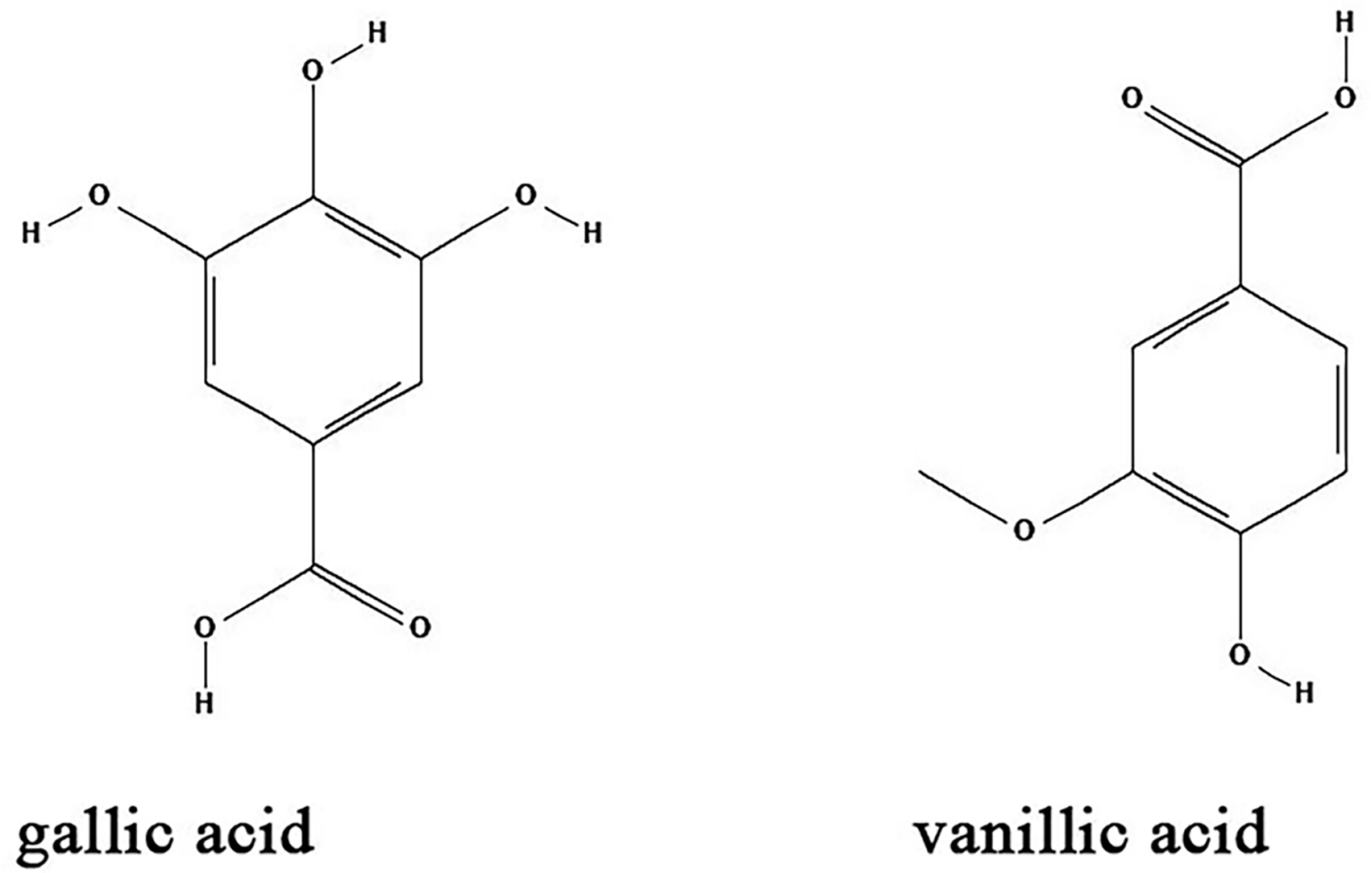
Chemical structures of the different organic acids that inhibit biofilm formation *via* QS. ChemDraw software has been utilized to draw the chemical structures of the molecules.

Natural organic acids are widely distributed in the leaves, roots and especially fruits of herbs such as umeboshi (pickled Japanese plum), schisandra (dry and mature fruit of *Schisandra chinensis*) and raspberry. Some natural organic acids have certain biological activities including antibacterial (Fontanay et al., 2008), anti-inflammatory (Wu et al., 2023), hypoglycemic (Pandey et al., 2022), antioxidant (Ma et al., 2018), and immune modulation (Wu et al., 2004; Fontanay et al., 2008; Ma et al., 2018). Common natural organic acids including gallic acid and vanillic acid have antibacterial biofilm effects (Table 1; Figure 5).

Gallic acid, also known as 3,4,5-trihydroxybenzoic acid, is a naturally occurring secondary metabolite. It is extracted from Green tea (*Camellia sinesis*) as a major component together with other anti-biofilm compounds such as EGCG, propyl gallate, and octyl gallate (Vidigal et al., 2014). The anti-biofilm activity of gallic acid has been investigated in diverse bacteria species. Gallic acid and ethyl gallate extracted from *Libidibia ferrea* (Mart. ex Tul.) inhibits *Streptococcus pyogenes* biofilms by downregulation of the expression of *gtfB*, *gtfC* and *gtfD* genes (Passos et al., 2021). Gallic acid at a concentration of 2 mg/ml significantly inhibits the expression of *pgaABCD* genes and effectively suppress the formation of *E. coli* biofilm in a dose-dependent manner (Kang et al., 2018). Moreover, high concentrations of gallic acid inhibited the biofilm formation and growth of *Proteus* spp., *Pseudomonas* spp., *Salmonella* spp., *Streptococcus mutans*, and *S. aureus* (Albutti et al., 2021). *In vivo*, gallic acid reduces inflammation and proliferation of *Brucella abortus* in spleens of mice (Reyes et al., 2018). Vanillic acid is a benzoic acid derivative that can be extracted from vanilla beans. Studies showed that vanillic acid inhibited the QS-dependent violacein biosynthesis in *C. violaceum* and biofilm formation in *Aeromonas hydrophila* by downregulation of AHL production (Deryabin et al., 2019). However, the detailed mechanisms of vanillic acid on biofilm formation needs to be further elucidated.

### Alkaloids

**Figure 6:**
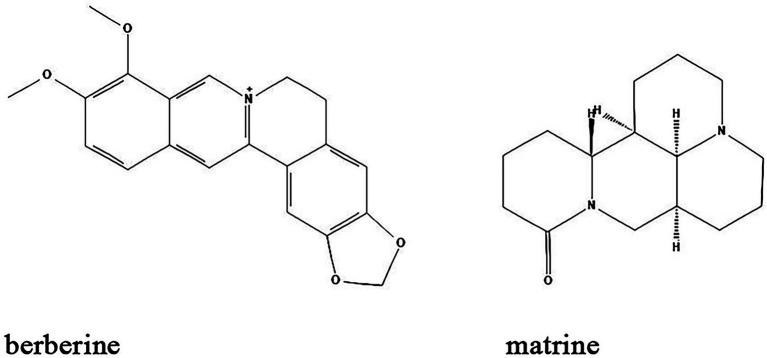
Chemical structures of the different alkaloids that inhibit biofilm formation *via* QS. ChemDraw software has been utilized to draw the chemical structures of the molecules.

Alkaloids are nitrogen-containing heterocyclic compounds which are widely found in plants including *Papaveraceae*, *Berberidaceae*, and *Fabaceae*. Lots of alkaloids have been identified so far and many of them exert antibacterial effects with broad spectrum and fewer adverse effects (Table 1; Figure 6). Their main antibacterial mechanisms include (i) inhibition of bacterial cell wall synthesis; (ii) inhibition of bacterial biofilm formation; (iii) alteration of cell membrane permeability; (iv) inhibition of bacterial metabolism; and (v) inhibition of nucleic acid and protein synthesis (Larghi et al., 2015; Table 1; Figure 5).

Berberine is an alkaloid extracted from *Coptis chinensis* and also an isoquinoline derivative according to its chemical structure. Berberine has been reported to have antibacterial efficacy in eliminating bacterial and fungal biofilms. As such, berberine exerted synergistic effects on inhibiting *Candida albicans*/*S. aureus* dual strain biofilms in combination with amphotericin B, an efficient antibiotic that utilized for the treatment of fungal infections in clinic (Gao et al., 2021). A study by Ning et al. demonstrated that berberine inhibited biofilm formation *via* downregulation of the expression of the QS regulatory gene *agrA* in a concentration-dependent manner in *S. aureus* (Ning et al., 2022). Moreover, Ferrazzano et al. found that berberine exerted efficient antimicrobial efficacy against diverse endodontic pathogens including *Fusobacterium nucleatum*, *Prevotella intermedia*, and *Enterococcus faecalis* (Ferrazzano et al., 2011). Interestingly, berberine also regulates gut microbiota and microbial tryptophan catabolites to protect mice from inflammatory bowel diseases (Zhang et al., 2019; Jing et al., 2021).

Matrine is another alkaloid that is widely distributed in *Sophora alopecuroides* (a perennial leguminous herb distributed in northwestern and northern China), broad bean roots and *Sophora flavescens*. It has anti-inflammatory, antibacterial, antioxidant, immunomodulatory and anticancer effects (Sun et al., 2022). Similar to berberine, matrine is also found to inhibit the biofilm formation of different bacteria species. Matrine reduce the formation of antimicrobial-resistant *E. coli* (a strain that showed resistant to different antibiotics) biofilms by downregulation of QS-related genes *luxS*, *pfS*, *sdiA*, *hflX*, *motA* and *fliA* (Sun et al., 2019). In *S. epidermidis*, the biofilm formation is also inhibited by matrine through decreasing the QS signaling molecule AI-2 activity (Jia et al., 2019). In combination with antibiotics, matrine dramatically decreases the multidrug-resistant *P. aeruginosa* biofilms (Pourahmad Jaktaji and Koochaki, 2022). Moreover, *in vivo* studies found that matrine can modulate the composition and functions of gut microbiota to improve gut barrier integrity and reduce murine colitis (Yao et al., 2021).

### Others

**Figure 7:**
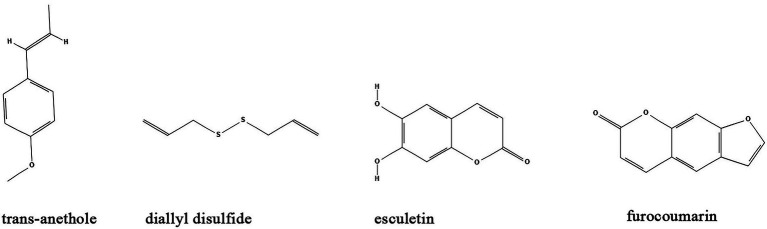
Chemical structures of other compounds that inhibit biofilm formation *via* QS. ChemDraw software has been utilized to draw the chemical structures of the molecules.

Besides the major classes of anti-biofilm compounds mentioned above, many other compounds have been identified from natural sources or TCMs with anti-biofilm activity including but not limited to trans-anethole, diallyl disulfide, esculetin, and furocoumarins. (Table 1; Figure 7).

Trans-anethole, the main component of anise oil, exhibit inhibitory effect on biofilm formation and the expression of QS-regulated virulence factors in *P. aeruginosa* by binding to LasR regulatory protein (Hançer Aydemir et al., 2018). Similarly, the *P. aeruginosa* biofilms and virulence factors including exonuclease LasA, elastase LasB, lectins LecA and LecB can also be inhibited by diallyl disulfide, a compound utilized in garlic oil (Li et al., 2018). Moreover, diallyl disulfide had beneficial effects on establishment of microbiota biofilms and colonic mucus production that alleviate murine colitis (Motta et al., 2015). Coumarins are a class of organic compounds which are not only isolated from sieve bean, but also in many different plants, such as Tonka beans, verbena, wild vanilla and orchid (ElNaggar et al., 2022). Studies found that some coumarins including esculetin and furocoumarins have broad range anti-biofilm activity by disturbing QS in *S. aureus*, *E. coli*, *S. typhimurium*, and *P. aeruginosa via* reduction of AHL (Girennavar et al., 2008; Dürig et al., 2010). Further studies demonstrated that esculetin is structurally compatible with the TraR AHL-binding site and downregulates numerous genes associated with QS signaling (Zeng et al., 2008; Zhang et al., 2018).

Apart from these CHMs metabolites, the anti-biofilm activities of some plant’s crude extract have also been investigated. For example, halogenated furanone compounds extracted from red seaweed *Delisea pulchra* can inhibit colonization, swarming and biofilm formation of Gram-negative bacteria, attenuate bacterial virulence and prevent bacterial infections (Chang et al., 2019; Aburto-Rodríguez et al., 2021). The structure of halogenated furanones is similar to that of the signaling molecule AHL, which compete with AHL for the receptor protein and replace AHL molecules binding to the receptor (Rabin et al., 2013). In *Vibrio fischeri* and *Vibrio harveyi*, halogenated furanones are also found to accelerate the folding of *luxR*, which in turn diminishes the ability of LuxR to bind to DNA and the transcription initiation process (Reuter et al., 2016). Moreover, Siddiqui et al. demonstrate that *Piper betle* extract (PBE) inhibit *P. aeruginosa* biofilm formation by reduction of AHL and EPS (Siddiqui et al., 2012). Also, PBE can reduce the virulence of *P. aeruginosa* by affecting the QS system (Datta et al., 2016).

## Nucleotide-based second messengers

Nucleotide-based second messengers are small non-protein molecules produced intracellularly. Bacteria can respond to extracellular signals through changes in the concentration of second messenger molecules (increase or decrease) by binding to cell surface receptors, regulating the enzymatic activity of intracellular metabolic systems, amplifying the original signal and thus inducing intracellular expression of a series of specific genes, and ultimately affecting a variety of physiological and biochemical processes in bacteria (Römling et al., 2013; Opoku-Temeng et al., 2016). Second messenger molecules have been shown to be involved in regulating bacterial growth and metabolism and other physiological functions, such as virulence factor expression (Ahmad et al., 2011, 2013), fatty acid synthesis (Zhang et al., 2013; Gerhardt et al., 2020; Li et al., 2022), cell wall metabolic homeostasis (Witte et al., 2013; Commichau et al., 2018), extracellular polysaccharide synthesis and biofilm formation (da Aline Dias et al., 2020; Junkermeier and Hengge, 2021). Six major types of second messengers have been discovered in bacteria so far, including c-di-GMP (Römling et al., 2013), c-di-AMP (Peng et al., 2016), cGAMP (Davies et al., 2012; Li et al., 2019), cGMP (Linder, 2010), cAMP (Harman, 2001) and (p)ppGpp (van Delden et al., 2001). While c-di-GMP is recognized as an ubiquitous second messenger for the regulation of bacterial biofilm formation, biofilm formation regulated by the other second messengers is only found in certain bacteria species, including *S. mutans* (Lemos et al., 2004; Peng et al., 2016), *Bacillus subtilis* (Gundlach et al., 2016; Townsley et al., 2018), *S. aureus* (Gries et al., 2016), *P. aeruginosa* (Luo et al., 2015), *K. pneumoniae* (Ou et al., 2017), and *E. coli* (Hufnagel et al., 2016; Li et al., 2019). Therefore, the development of novel anti-biofilm agents in terms of nucleotide-based second messengers is mainly targeted on c-di-GMP signaling.

### TCMs that inhibit second messenger molecules

**Figure 8:**
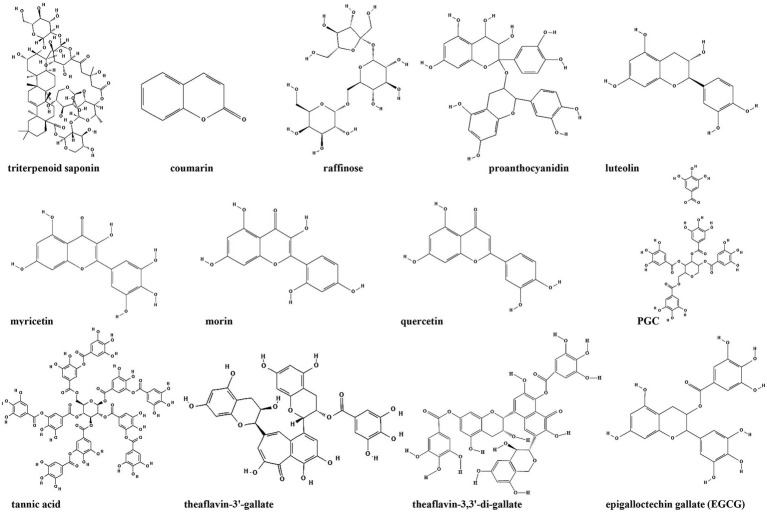
Chemical structures of the compounds that inhibit biofilm formation *via* second messengers (−related) signaling pathways. ChemDraw software has been utilized to draw the chemical structures of the molecules.

Given the important role of second messenger-regulated signaling pathways in bacterial biofilm formation, the development of antimicrobial compounds *via* second messenger-regulated signaling pathways to control infections has become a research priority. The mechanism of action for blocking second messenger signaling is broadly divided into three categories: (i) inhibition or activation of second messenger synthases; (ii) inhibition or activation of second messenger degradation enzymes; and (iii) competition for signaling pathway receptor proteins (Zhou et al., 2013; Sambanthamoorthy et al., 2014; Zheng et al., 2016). Although thousands of literatures have provided biological insights into second messenger signaling so far, the development of small-molecule inhibitors of second messengers on bacterial biofilm formation is significantly lagging behind, with even fewer studies on natural compounds such as TCMs metabolites as inhibitors (Opoku-Temeng et al., 2016). Compounds which inhibit bacterial second messengers signaling are listed in Table 2; Figure 8.

**Table 2:** Different classes of anti-biofilm TCMs metabolites and their mechanisms of action *via* bacterial second messengers (−related) signaling pathways.

TCMs metabolites	Main plant origin	Mechanism of action	Target bacteria	Reference
**Terpenoids**				
Triterpenoid saponin	*Pisum sativum*	Inhibits DGC	*Acetobacter xylinum*	Ohana et al. (1998)
**Flavonoids**				
Luteolin	Mignonette	Inhibits the assembly of amyloid curli fibers by driving CsgA subunits into oligomers	*E. coli*	Pruteanu et al. (2020)
Myricetin	Red bayberry			
Morin	Morus flavescens			
Quercetin	*Usnea longissimi*			
**Phenols**				
Proanthocyanidin	Grape seeds	Modulation of the intracellular c-di-GMP level	*Vibrio cholerae*	Pederson et al. (2018)
Tea polyphenols	Green tea (*Camellia sinesis*)	Downregulates c-di-AMP level	*E. coli*; *Bacillus subtilis*	Opoku-Temeng and Sintim (2016)
theaflavin-3′-gallate	Green tea (*Camellia sinesis*)		*B. subtilis*	
theaflavin-3,3′-di-gallate	Green tea (*Camellia sinesis*)		*B. subtilis*	
**Others**				
Catechol-containing sulfonyl hydrazide	*Acacia catechu (L.f.)Willci.*	Inhibits DGC PleD	*C. crescentus*	Fernicola et al. (2016)
Counarins	Tonka Beans; verbena; wild vanilla and orchid	Alters the expression of genes associated with the type III secretion system and c-di-GMP metabolism	*P. aeruginosa*	Zhang et al. (2018)
Raffinose	Ginger	Decreases the concentration of c-di-GMP by increasing the activity of c-di-GMP-specific phosphodiesterase	*P. aeruginosa*	Kim et al. (2016)
PGG	Green tea (*Camellia sinesis*)	Interferes with initial attachment and the synthesis of polysaccharide intercellular adhesin	*S. aureus*	Lin et al. (2011)

TCMs, Traditional Chinese medicines; DGC, Diguanylate cyclase; PGG, 1,2,3,4,6-Penta-O-galloyl-β-D-glucopyranose; c-di-GMP, Cyclic dimeric guanosine monophosphate; c-di-AMP, Cyclic dimeric adenosine monophosphate; cAMP, Cyclic adenosine monophosphate.

Cyclic di-GMP is recognized as an ubiquitous second messenger that regulates bacterial sessility/motility lifestyle transition (Simm et al., 2004), cell cycle (Xu et al., 2020), virulence (Ahmad et al., 2011), biofilm formation and dispersal (Ross et al., 1987; Miller et al., 2022). The intracellular concentrations of c-di-GMP depend on the rates of synthesis and degradation, which are regulated by diguanylate cyclase (DGC) and phosphodiesterase (PDE), respectively, that can sense different signals (Boyd and O’Toole, 2012; Römling et al., 2013). At present, c-di-GMP signaling inhibitors discovered in herbs are mostly c-di-GMP analogs or non-nucleotide small molecules that inhibit DGCs. Ohana et al. isolated a specific and efficient inhibitor of DGC, triterpenoid saponin, from extracts of *Pisum sativum*. Triterpenoid saponin inhibits DGC of *Acetobacter xylinum*, thereby reduces the intracellular concentration of c-di-GMP (Ohana et al., 1998). *In vivo*, triterpenoid saponin increase beneficial bacteria, while decreases sulfate-reducing bacteria, and alleviate intestinal inflammatory gut environment in mice (Chen L. et al., 2016). Moreover, using a virtual approach with a three-dimensional pharmacophore model, two catechol-containing sulfonyl hydrazide compounds are identified with the ability to competitively inhibit DGC PleD in *Caulobacter crescentus* and could serve as potential inhibitors of bacterial c-di-GMP signaling (Fernicola et al., 2016).

Coumarin is found in tonka beans, verbena, wild vanilla and orchid, and has the smell of fresh hay and fenugreek (ElNaggar et al., 2022). Coumarins have been shown to have antibacterial activity as a QSI in a broad spectrum of pathogens. Coumarin alters the expression of genes associated with the type III secretion system and c-di-GMP metabolism to inhibit biofilm formation. Coumarin significantly reduces the cellular c-di-GMP levels of *P. aeruginosa* PAO1 and clinical *P. aeruginosa* strains (Zhang et al., 2018). Raffinose, a plant galactose derived from ginger, can bind to a carbohydrate-binding protein LecA to effectively inhibit *P. aeruginosa* biofilm and alter bacterial phenotype without impairing bacterial growth (Kim et al., 2016). In addition, raffinose also decreases the concentration of c-di-GMP by increasing the activity of c-di-GMP-specific phosphodiesterase (Kim et al., 2016). Moreover, procyanidins are the general name of a large class of polyphenol compounds, which are abundant in grape seeds. Water-soluble extract from cranberry standardized to 4.0% proanthocyanidins could significantly inhibit *Vibrio cholerae* biofilm formation by reducing the biofilm matrix production and secretion *via* modulation of the intracellular c-di-GMP level (Pederson et al., 2018).

Besides the compounds mentioned above, it’s demonstrated that green tea polyphenol EGCG inhibits *E. coli* biofilms by elimination of the biofilm matrix *via* interfering with CsgD expression and the assembly of curli subunits into amyloid fibers (Serra et al., 2016). Study from the same group also identified several plant flavonoids including luteolin, myricetin, morin and quercetin as biofilm inhibitors. These flavonoids strongly reduce the extracellular matrix production by directly inhibiting the assembly of amyloid curli fibers through driving CsgA subunits into oligomers (Pruteanu et al., 2020). Additionally, 1,2,3,4,6-Penta-*O*-galloyl-β-D-glucopyranose (PGG), an active ingredient in plants, inhibits *S. aureus* biofilm formation by interfering with initial attachment and the synthesis of polysaccharide intercellular adhesin (Lin et al., 2011), but whether c-di-GMP is also involved in this process stills unknown.

Plant anti-biofilm compounds targeting other second messengers are quite few and still needs to be discovered. Opoku-Temeng et al. identified three tea polyphenols including tannic acid, theaflavin-3′-gallate and theaflavin-3,3′-di-gallate as c-di-AMP inhibitors in *B. subtilis*. They found that these polyphenols specifically inhibited DisA activity to downregulate c-di-AMP level (Opoku-Temeng and Sintim, 2016).

## Conclusion

The majority of bacteria in nature live in a biofilm state, and infections due to biofilms pose a great threat to clinical treatment. The bacterial QS system and second messenger signaling pathways play an important role in the regulation of biofilm formation, but their complex regulatory mechanisms need to be further investigated. These works on bacterial biofilm formation have provided many potential therapeutic targets for the development of antibacterial drugs. Many TCMs from natural compounds are well-known for their safety and less toxicity to host (Flower et al., 2015; Liao et al., 2022). The different chemical classes of TCMs metabolites with antibacterial activity act in the QS system and second messenger signaling pathways mainly by reducing the production of signaling molecules or competing for receptor proteins, and no TCMs’ metabolites with enzymatic activity to degrade signaling molecules have been discovered. In addition, most TCMs’ metabolites work alone at high concentrations and take a long time to function without the ability to kill bacteria, but they work well in combination with antibiotics or as antibiotic potentiators. Strategies such as modification of chemical structures and precision delivery by nanomaterials to the target of action can be developed to enhance the antibacterial ability of TCMs’ metabolites. In conclusion, with the continuous development of life science, TCMs, as a valuable asset left to mankind by nature and our ancestors, must have a longer-term development prospect in the fight against bacterial infections.

## Author contributions

FL and JG conceived and designed the manuscript. MZ wrote the draft of the manuscript. CQ, QJ, and JD prepared the figures and edited the tables. FL compiled and reviewed the draft of the manuscript. LL, WH, and JG co-administrated the project. All authors contributed to the article and approved the submitted version.

## Funding

This work was financially supported through grants from the National Natural Science Foundation of China (grant no. U19A2038, 31872505, 32072824, and 32102670), the Natural Science Foundation of Jilin Province (grant nos. 20200201120JC, 20220101295JC), the Fundamental Research Funds for the Central Universities, Shandong Provincial Modern Agricultural Industry Technology System (SDAIT-27), and Key Technology Research and Development Program of Shandong (2021TZXD012).

## Conflict of interest

The authors declare that the research was conducted in the absence of any commercial or financial relationships that could be construed as a potential conflict of interest.

## Publisher’s note

All claims expressed in this article are solely those of the authors and do not necessarily represent those of their affiliated organizations, or those of the publisher, the editors and the reviewers. Any product that may be evaluated in this article, or claim that may be made by its manufacturer, is not guaranteed or endorsed by the publisher.
